# B-cell lymphoma 2 family members and sarcomas: a promising target in a heterogeneous disease

**DOI:** 10.37349/etat.2023.00154

**Published:** 2023-08-24

**Authors:** Rui Caetano Oliveira, João Gama, José Casanova

**Affiliations:** Regina Elena National Cancer Institute, Italy; ^1^Centro de Anatomia Patológica Germano de Sousa, 3000 Coimbra, Portugal; ^2^Coimbra Institute for Clinical and Biomedical Research (iCBR), 3000 Coimbra, Portugal; ^3^Centre of Investigation on Genetics and Oncobiology (CIMAGO), 3000 Coimbra, Portugal; ^4^Pathology Department, Centro Hospitalar e Universitário de Coimbra, 3000 Coimbra, Portugal; ^5^Orthopedic Oncology Department, Centro Hospitalar e Universitário de Coimbra, 3000 Coimbra, Portugal; ^6^Faculdade de Medicina da Universidade de Coimbra, 3000 Coimbra, Portugal

**Keywords:** B-cell lymphoma 2, sarcomas, therapy

## Abstract

Targeting the B-cell lymphoma 2 (Bcl-2) family proteins has been the backbone for hematological malignancies with overall survival improvements. The Bcl-2 family is a major player in apoptosis regulation and, has captured the researcher’s interest in the treatment of solid tumors. Sarcomas are a heterogeneous group of diseases, comprising several entities, with high morbidity and mortality and with few specific therapies available. The treatment for sarcomas is based on platinum regimens, with variable results and poor outcomes, especially in advanced lesions. The high number of different sarcoma entities makes treatment standardization as well as the performance of clinical trials difficult. The use of Bcl-2 family members modifiers has revealed promising results in *in vitro* and *in vivo* models and may be a valid option, especially when used in combination with chemotherapy. In this article, a revision of these results and possibilities for the use of Bcl-2 family members inhibitors in sarcomas was performed.

## Introduction

Cancer is a heterogeneous group of diseases characterized by high proliferation, microenvironment modulation, and breakthrough of physical barriers, and evasion of programmed cell death—apoptosis. Apoptosis is essentially a subtype of programmed cell death, fundamental in several physiological and pathological events, eliminating damaged cells [1].

There are two pathways for apoptosis: an extrinsic pathway, mediated by ligands and cell surface death receptors, and an intrinsic pathway, that results from mitochondrial processes [2].

One of the main regulators of the intrinsic pathway is the B-cell lymphoma 2 (Bcl-2) family of proteins. Recent pharmaceutical developments have explored this pathway as a treatment option, with considerable success [3].

Sarcomas are rare tumors, usually detected in bone and soft tissues, but which have high morbidity and mortality. They comprise more than 50 histological subtypes, prompting additional difficulty for treatment standardization and homogenous therapy [4]. This heterogeneity of sarcomas makes the performance of clinical trials and quality of life evaluation even more difficult [5, 6].

Sarcomas have origin in several genetic alterations, such as fusion proteins, tumor suppressor and cell cycle regulatory gene mutations, and DNA copy-number variations. These changes can be grouped in sarcomas with simple karyotypes and specific genetic alterations [such as EWS RNA binding protein 1 (*EWSR1*) fusions in Ewing sarcoma, paired box 3 (*PAX3*) fusions in alveolar rhabdomyosarcomas, among others] and in sarcomas with complex genetic modifications [7].

Due to this genetic heterogeneity, especially in the setting of advanced disease, therapies are limited, usually based on chemotherapy, with a low overall survival [8]. Due to resistance to monotherapies, a combined treatment with doxorubicin and ifosfamide (core treatment in osteosarcoma [9] and in some histological subtypes of soft tissue sarcoma such as leiomyosarcoma [10]) is usually employed to overcome resistance, but with only a mild improvement in overall survival and with high toxicity reports [11].

In this review, there is a focus on the Bcl-2 functions in cancer, with an emphasis on sarcomas, and their role in precision therapy, mainly of the Bcl-2 homology 3 (BH3)-mimetics/Bcl-2 inhibitors.

## The Bcl-2 family

This family is essentially defined by its founder member, the *BCL-2* gene located at chromosome 18, which was first identified by chromosomal analysis of follicular lymphoma [12]. In follicular lymphoma, the translocation t(12;18) leads to a constitutive *BCL-2* expression, which promotes oncogenesis cell death resistance [13]. After this discovery, more than 15 proteins have been added to this family, each with one or more BH domains [3].

The family of Bcl-2 proteins controls the cell death mechanism by regulating the mitochondrial outer membrane permeability, which induces the irreversible release of intermembrane space proteins with caspase activation and apoptosis [14].

Further studies allowed the subclassification into anti-apoptotic [BCL-2, Bcl-extra large (BCL-xL), Bcl-2-like protein 2 (BCL-W), induced myeloid leukemia cell differentiation protein (MCL-1), Bcl-2-related protein A1 (BFL-1/A1)] and pro-apoptotic members. The pro-apoptotic Bcl-2 family members can be further subdivided into multi-BH domains proteins, which contain BH1, BH2 and BH3 domains [Bcl-2-associated X protein (BAX), Bcl-2 homologous antagonist/killer (BAK), Bcl-2 related ovarian killer (BOK)], and BH3-only members [BCL2 associated agonist of cell death (BAD), BH3 interacting domain death agonist (BID), Bcl-2-interacting killer (BIK), Phorbol-12-myristate-13-acetate-induced protein 1 (NOXA), p53 upregulated modulator of apoptosis (PUMA)] [14]. This affects the molecular structure, with the BH3-only members exhibiting a less structured conformation when in solution (except for BID) and the remaining members having a highly conserved tertiary structure [15].

This is associated with distinct functions, and currently, there are two (non-exclusive) models: a direct model where the BH3-only proteins can directly activate Bax and Bak, which are pro-apoptotic, and can be hampered by anti-apoptotic proteins [16]; and an indirect model, where BH3-only proteins challenge the inactive dimerization between anti and pro-apoptotic proteins [17]. The indirect model is very interesting because when Bax and Bak are released from their interaction with anti-apoptotic proteins, they are already in an active state and do not require further activation by BH3-only proteins. This mechanism is the main reason why BH3-mimetics are more efficient in inducing cell death in cells that have a high expression of antiapoptotic proteins, such as cancer cells [18].

Both mechanisms can coexist, but regardless of the mechanism, the result is the oligomerization and conformation changes in Bax and Bak, with the formation of pores in the mitochondrial outer membrane, cytochrome c release, caspase activation and apoptosis induction [19–22].

In physiological conditions, there is an equilibrium between the pro-survival and pro-apoptotic molecules. In cancer, this balance is disrupted, with a shift towards a pro-survival state. The pro-survival proteins (BCL-2 family proteins such as Bcl-2 and Mcl-1) sequester the BH3-only members, preventing them from activating the effectors Bax, Bok and Bak and, therefore, neutralizing the formation of pores in the mitochondria membrane and consequent initiation of apoptosis [23, 24]. As explained before, the use of BH3 mimetic drugs may target Bcl-2. Bcl-xL and Mcl-1, freeing the BH3-only proteins to initiate apoptosis, would be of high therapeutic value.

**Figure 1:**
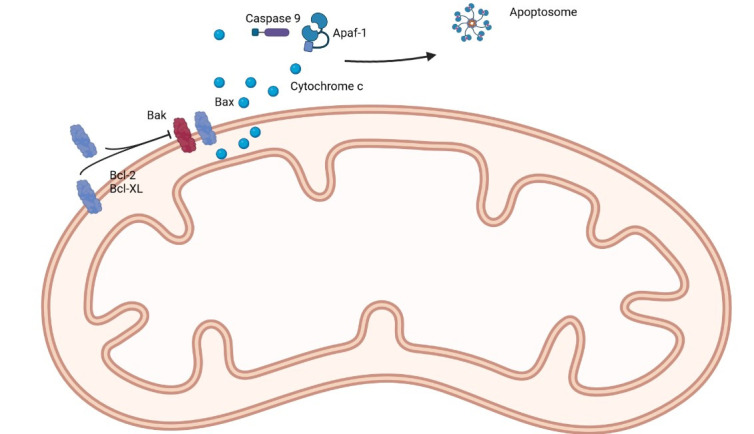
A simple representation of the main proteins of the Bcl-2 family. Apaf-1: apoptotic protease activating factor 1. Created in BioRender™ (https://www.biorender.com/)

The first selective Bcl-2 inhibitor was navitoclax (targets Bcl-2, Bcl-xL, and Bcl-W), followed by Obatoclax (targets Bcl-2, Bcl-xL, Mcl-1, and Bcl-W) and venetoclax (selectively targets Bcl-2), with the latter being (probably) the most well-known, due to its success, and favorable safety profile, in the treatment of chronic lymphocytic leukemia, acute myeloid leukemia, some subtypes of lymphoma and multiple myeloma [25]. A simple representation of the main proteins of the Bcl-2 family can be seen in Figure 1.

## Cancers with frequent Bcl-2 alterations

Considering the functions of the Bcl-2 family of proteins is not surprising that they have increased expression in cancer. Evasion of apoptosis is a fundamental process in oncogenesis [26]. This upregulation is found because of several mechanisms, with chromosomal translocation, gene amplification, and increased gene expression/translation as the most common ones.

Bcl-2 translocation is frequently found in follicular lymphoma, as previously mentioned, but is also expressed in other hematologic malignancies such as lymphoma of mucosa-associated lymphoid tissue [27], diffuse large Bcl [28] and acute myeloid leukemia [29]. The Bcl-2 translocation results in overexpression of the Bcl-2 protein, and therefore in the inhibition of the apoptosis [30]. In these hematological malignancies, the Bcl-2 family alterations have been pointed out as new therapeutic possibilities, with the use of selective Bcl-2 inhibitors such as venetoclax, among others [25]. However, the use of these drugs in hematology is not the focus of this review.

Following the studies in hematologic malignancies, many studies have found amplifications of Bcl-2 family genes, such as *MCL1* and *BCL2L1* (responsible for encoding Bcl-xL) in solid tumors, such as lung, breast, uterine and bladder cancer, which was confirmed by The Cancer Genome Atlas data [3]. These findings have paved the way for clinical trials in lung cancers with navitoclax, a novel Bcl-2 family inhibitor, with interesting results [31].

Studies with colorectal cell lines have found the anti-tumor effect of Bcl-xL inhibitor [32], the same happening in studies with lung cancer cell lines [33]. In a recent study in breast cancer, there was a role of Bcl-xL in cell migration and mitochondrial metabolism, favouring the onset and dissemination of metastases, prompting for modulation of the VDAC1/Bcl-xL interaction as a promising target for anti-tumor therapy in the context of metastatic breast cancer [34]. Bcl-xL inhibitors (A-1155463 and A-1331852, for example) have emerged as a very interesting option, since they appear to have the potential to enhance the efficacy of docetaxel in solid tumors and avoid the exacerbation of neutropenia observed with navitoclax (because inhibits both Bcl-xL and Bcl-2) [35]. Bcl-xL inhibitors are known for their platelet toxicity, but this can be minimized by the adoption of lead-in dosing schemes or by the preselection of thrombocytopenia low-risk patients [36]. Besides the reduction of dose when using a Bcl-xL inhibitor, a key study that investigated the neutropenia induced by navitoclax, showed that this was mainly driven by the Bcl-2 inhibition, and not by the Bcl-xL one [35, 37].

A very intersecting study in 2020 by Scherr et al. [38] found by RNA expression, in more than 1,500 colorectal cancers, that Bcl-xL was the only overactivated antiapoptotic Bcl-2 protein. Using colorectal cancer cells, they were able to demonstrate that the use of WEHI-539 (a Bcl-xL inhibitor) was able to induce apoptosis, and after Bcl-xL knockdown, there was an increase in the response to irinotecan and 5-fluorouracil (5-FU). Still, in the same study, the authors translated those findings to animal models, in which they found that the addition of the Bcl-xL inhibitor to 5-FU caused the strongest anti-tumoral effect, proving support for association therapies. Several studies with a focus in tumor cells state that a Bcl-xL inhibitor as a single agent is not adequate to promote apoptosis [39]. There are reports in cancer cells and animal models, that the use of BTSA1.2 (an orally bioavailable Bax activator) in combination with Bcl-xL has a synergist anti-tumoral effect while being safe for healthy tissues [39, 40].

This mechanism in which solid tumors rely on Bcl-xL for survival has led to several studies focusing on selective Bcl-xL inhibitors [41]. We must also integrate the “non-apoptotic” role of Bcl-xL, namely its mechanism of action for tumor survival, non-related with BH3 and Bcl-2 directly, which should be referred to. Bcl-2 family proteins have a role in mitochondrial structure and functions. Bcl-xL is associated with mitochondrial fission and synaptic activity [42], and it was associated with a different apoptotic function: instead of the death of the entire cell, only a small appendage of a neuron would die, in an event so-called normal synaptic plasticity [41]. What’s more curious in these interactions is that on a mouse model with a mutated Bcl-xL, unable to be cleaved, the cells were more resistant to an ischemic event, which is the opposite of its expected function in killing tumor cells [43]. Bcl-xL is also implicated in the calcium homeostasis, more specifically with an increase of calcium leak in the endoplasmic reticulum by direct binding to the inositol 1,4,5-trisphosphate (IP_3_) receptors, inducing a low basal calcium concentration in the reticulum, and thus a reduction in stress-mediated calcium release [44, 45].

Therefore, the non-canonical functions of this family of proteins have also to be considered in order to fully understand their biological properties and therapeutic potential, making this a very intriguing puzzle.

## Sarcomas with regular Bcl-2 expression

There are sarcomas that are usually associated with Bcl-2 expression, namely on immunohistochemistry. This section will describe them and the possibilities for Bcl-2 targeted therapy.

### Synovial sarcoma

Synovial sarcoma (SS) is a sarcoma most often diagnosed in teenagers and young adults. It is highly aggressive, and the standard treatment is chemotherapy with variable results. Nowadays, the diagnosis is based on the finding of the *SS18-SSX* gene fusion, but in the past, a high expression of Bcl-2 by immunohistochemistry was a core element [7]. Since the gene fusion is not a viable target, the modulation of Bcl-2 expression via inhibitor-based therapies can be a valuable option. However, despite the expression of Bcl-2 in SS, the targeting of Bcl-2 has not been successful in preclinical trials with venetoclax [46].

A recent study has confirmed the high levels of Bcl-2 in SS [47] but found very low levels of *NOXA*, the endogenous inhibitor of MCL-1. Due to that low level of *NOXA*, *MCL-1* was not regulated and, expressed its anti-apoptotic function, resulting in high levels of Bcl-2.

Using cell lines and patient-derived xenografts, the authors demonstrated that low levels of *NOXA* were associated with resistance to venetoclax, similar to what happens in hematologic cancer, inducing an MCL-1 mediated resistance [48]. Using vector methodology, they established a SS cell line with high *NOXA* expression and assessed the sensitivity of the tumor cells to venetoclax-mediated cell death, since *NOXA* specifically binds with MCL-1 and inactivates it [14]. The study demonstrated a synergistic effect of the combined inhibition of Bcl-2 and MCL-1. The inhibition of MCL-1 was able to increase the sensitivity of tumor cells to venetoclax (*in vitro*) and induced tumor regression *in vivo* with the same agent. The mechanism is that inhibition of MCL-1 allows liberation of pro-apoptotic factors, and consequently the induction of apoptosis [47].

Interestingly, one tumor cell line, defined as atypical, without *SS18-SSX* fusion and Bcl-2 expression was resistant to the combination therapy. These findings have a high potential for clinical translation and are in line with other studies that state that the Bcl-2 protein level is not enough for assessing the sensitivity of cancer cells to venetoclax and, MCL-1 and Bcl-xL proteins have a pivotal role in antagonizing the activity of venetoclax [49, 50]. Other resistance mechanisms are described in the literature, such as specific mutations in Bcl-2 (G101V and D103Y) and high resistance to venetoclax [51, 52] but also mutations in BAX (G179E), which cause a diminution in its binding capacity to the mitochondrial membrane, due to translocation to the mitochondria, and therefore preventing BAX-mediated apoptosis [53]. The G179E BAX mutation is also characterized by inducing cross-resistance (at least partial) to other anti-tumoral drugs [53].

Mutation in BAX (G179E) is defined by an inactive form, so in order to target Bax, this has to be activated [54]. There are several drugs available that activate Bax indirectly, but direct Bax activation can provide a specific anti-tumoral approach [54]. However, Bax is rather special, since it has unique and pivotal sites which are not shared by the other members of the Bcl-2 family, prompting mechanisms for a targeted approach with a desired outcome and less adverse effects [55]. Bax participates in both the intrinsic and extrinsic pathways of apoptosis, which does not happen with BH3-only proteins because they depend on Bax/Bak, so Bax activation would be of extreme importance for apoptosis promotion [56]. Many anti-tumoral therapies induce cell death by resorting to Bax activation, so a direct effect and activation of Bax could be also the key for combined therapies [54].

By inducing conformational changes in Bax, they will promote a change from inactive to active state, thus exerting its function. However, some physical constraints such as a negative charge within a hydrophobic alpha-helix, may be an obstacle [57]. Recently, serine 184 (S184) was identified as a critical switch for apoptotic activity control in Bax, and the use of a structural pocket about it was able to provide a docking site for several small molecules of Bax agonists [58]. In this research, Xin et al. [58] were able to develop three compounds—SBMA1, SBMA2, and SBMA3, which induced conformation changes by blocking S184 phosporilation and prevented the creation of a negative charge; Bax was then able to bind with the mitochondrial membrane, with oligomers formation, subsequent cytochrome c release, and apoptosis induction. The anti-tumoral effect was observed in lung cancer cells, but also in *in vivo* models without relevant toxicity in non-tumoral tissue. The activation of Bax directly by small molecules had also been done in 2012 by Gavathiotis et al. [59] but in non-tumoral cells—genetically modified mouse fibroblasts.

The study from Barrott et al. [46] also demonstrated that the specific inhibition of Bcl-2 had a minimal impact on SS. Still, the use of a Bcl-xL inhibitor showed an anti-tumoral activity both in cell lines and animal models, providing evidence that combined therapies could be the solution.

A recent study from Sobol et al. [60] also refers to the therapeutic effect of Bcl-xL inhibitors (ABT-263) as sensitizers for radiotherapy in SS. In this study, the authors found that cell lines treated with the anti-Bcl-xL showed major radiation-induced damage, even in radiation-resistant cell lines. The potential clinical application of these findings is vast.

**Figure 2:**
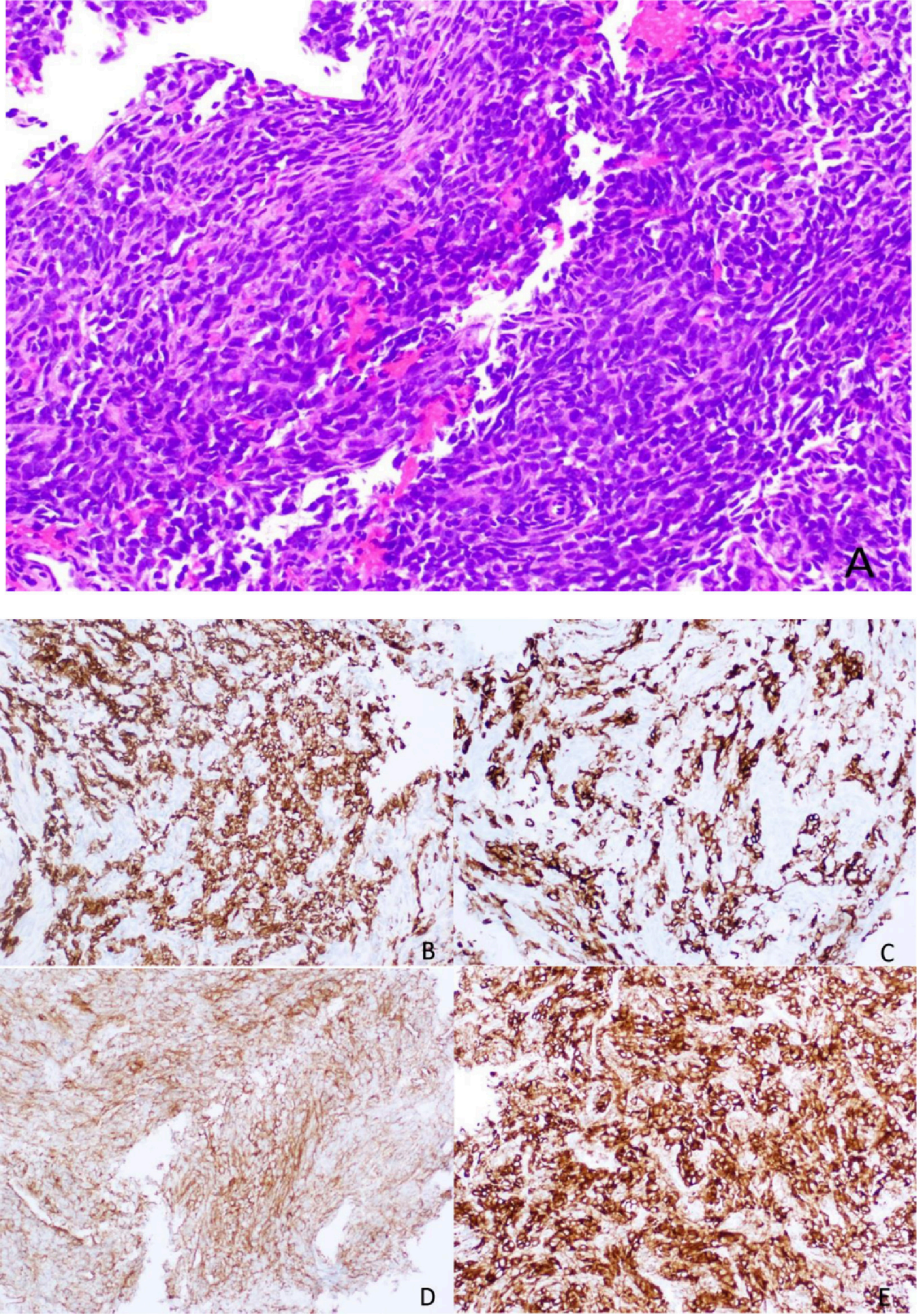
Histology of a SS, with a spindle cell morphology, sometimes with a herringbone pattern. The tumor cells are usually positive for Bcl-2 immunostaining (smaller image in the right lower corner, Figure 2E) *Note.* Reprinted from “Synovial sarcoma of bone: sarcoma typically of soft tissues presenting as a primary bone tumor,” by Caracciolo JT, Henderson-Jackson E, Binitie O. Radiol Case Rep. 2018;14:204–7 (https://www.sciencedirect.com/science/article/pii/S1930043318304692?via%3Dihub). CC BY-NC-ND.

The development of new Bcl-2 inhibitors with an affinity for different pro-survival Bcl-2 members and the use of combination therapies targeting also MCL-1 and Bax may be the answer to overcoming these resistances. The Figure 2 represents a SS with Bcl-2 expression.

### Solitary fibrous tumor

Originally described in the pleura, solitary fibrous tumor (SFT) has been described almost in every anatomical location [61]. Recently a *NAB2-*signal transducer and activator of transcription 6 (*STAT6*) gene fusion was discovered as a genetic hallmark of SFT, assessed by immunohistochemistry [62], facilitating the diagnosis, that until then was based on morphology, CD34, and Bcl-2 expression.

The current WHO Classification of Soft Tissue and Bone Tumors classifies SFT as a fibroblastic neoplasm with intermediate (rarely metastasizing) behavior [7] but some cases have been described with features that contribute to malignancy, namely: older age, larger tumor size, increased cellularity, increased mitotic activity (≥ 4/10 HPFs or > 2 mitoses/2 mm^2^), nuclear pleomorphism, tumor necrosis, and infiltrative borders [7, 63]. Interestingly, these features can be seen in recurrences and metastases of SFT that lacked malignant characteristics at the time of primary resection [64]. Several scores have been proposed [7, 65–69], with the one proposed by Demicco EG and colleagues [70] being considered as the most accurate in prognosis.

The Bcl-2 expression in SFT could prompt for targeted therapy, but due to the rarity of this tumor, there is a lack of randomized control trials and no consensus on treatment [71]. Chemotherapy is still the core of the treatment for malignant SFT in neo and adjuvant settings [72]. Anti-angiogenic treatment has been proposed in advanced and aggressive lesions with good results in clinical trials [73, 74], but no targeted Bcl-2 therapies have been described to date.

## Ewing sarcoma

Ewing sarcoma (ES) is a high-grade sarcoma, often diagnosed in children and young adults, which may occur both in soft tissue and bone [75]. Morphologically ES have a small, blue, and round cell appearance and are genetically defined by *EWSR1* fusion [76]. Currently, three categories are acknowledged: round cell sarcomas with *EWSR1* gene fusion with non-ETS family members, *CIC*-rearranged sarcomas, and *BCOR*-rearranged sarcomas [7].

The standard care for ES is intensive chemotherapy, neoadjuvant and adjuvant, with good overall survival—75% at five years; however, for patients with relapsed or metastatic disease after treatment, the survival drops to 30% [77]. The most frequent translocation event (in circa 90%) in ES is the *EWSR1-FLI1* t(11;22)(q24;q12), which activates the transcription factor FLI1 [78], but the FLI1 is currently undruggable.

In 2012, studies conducted by Brenner et al. [79] and Garnett et al. [80] demonstrated that ES with the *ESWR1-FLI1* translocation had a high sensitivity to poly ADP ribose polymerase (PARP) inhibitors, which was later confirmed by other groups [81–83]. Despite this promising data, the initial clinical studies were rather disappointing, without objective response to olaparib treatment [84].

To identify the resistance mechanisms, in 2019, a study conducted by Heisey et al. [85] resorting to cell lines and animal xenograft models, was able to demonstrate that the cell line with resistance to olaparib after chemotherapy had a high expression of Bcl-2, which was the responsible for the apoptotic resistance to olaparib. The addition of venetoclax, a Bcl-2 specific inhibitor, did not show sensitization of the tumor cells to olaparib, but the addition of navitoclax, a dual inhibitor of Bcl-2 and Bcl-xL, induced a sensitization of the tumor cells to the PARP inhibitor, with a marked loss of cell viability, even with low doses of olaparib, in two different cell lines, one chemoresistant and other chemosensitive. The experiments in the animal models replicate these findings, with a minimum effect of navitoclax and olaparib in a monotherapy regimen, but when used in combination, they demonstrated a robust inhibition of tumor growth, without augmented hematologic toxicity. Therefore, the co-targeting on Bcl-2 and Bcl-xL can sensitize the ES to olaparib treatment, which may be of benefit in chemotherapy-resistant cases, prompting the need for clinical trials.

A very recent study, from Pascual-Pasto et al. [86] also addressed the question of ES sensitivity to Nab-paclitaxel. Using patient derived xenografts, they were able to demonstrate that low expression of Bcl-2 (assessed by immunohistochemistry) was associated with a high efficacy of Nab-paclitaxel, therefore prompting the use of immunohistochemistry for selecting patients for this therapy.

## Pediatric sarcomas

Although rare, pediatric cancer is a major cause of death. Rhabdomyosarcoma is the most frequent sarcoma in children, but neuroblastoma, ES, and osteosarcoma are also common [7]. In haematological malignancies the use of venetoclax is already ongoing in clinical trials for relapsed, refractory or high-risk leukaemia [87, 88]. Based on the previous findings in ES, a study in 2020 by Kehr et al. [89] demonstrated that rhabdomyosarcoma, osteosarcoma, and neuroblastoma cell lines were co-dependent of Bcl-xL and MCL-1 for survival. The single treatment with Bcl-2, Bcl-xL, or MCL-1 inhibitors did not show a valid anti-tumoral response. Once again, the co-treatment with Bcl-xL and MCL-1 inhibitors demonstrated high cellular death induced by caspase-dependent apoptosis. The treatment facilitated the Bax/Bak complex formation and led to a shift in the interaction pattern of the BH3-only proteins BIM and NOXA, disrupting their interactions with Bcl-xL and MCL-1. The co-treatment also displayed anti-tumor activity in the *in vivo* model of rhabdomyosarcoma (embryonic chicken model). Previously, in 2018, Faqar-Uz-Zaman et al. [90] had already stated that BH3 mimetics could be used to increase chemotherapy sensitization in rhabdomyosarcoma, findings that were also stated by Alcon et al. [91] in 2020. This study from 2020 describes that combining BH3 mimetics with conventional chemotherapy (vincristine and doxorubicin) bypasses tumor resistance, with an increase in treatment efficiency and less secondary effects being reported. A very interesting, phase I study, is ongoing, studying the effect of venetoclax added to chemotherapy in pediatric and young adult patients with any relapsed/refractory tumor type with evidence of BCL-2 expression, which will present additional data to this subject [92].

**Figure 3:**
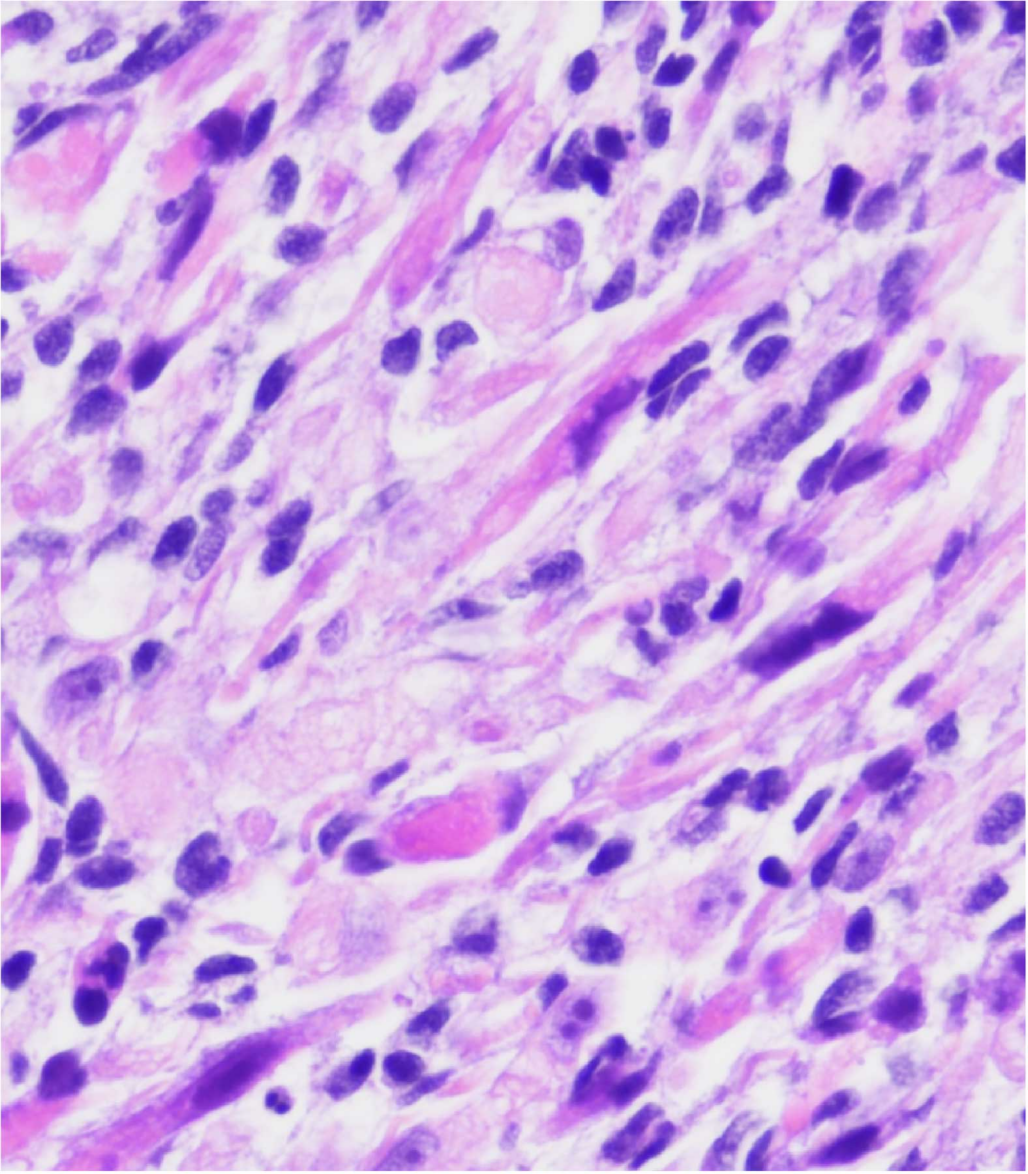
A rhabdomyosarcoma from the orbit with considerable atypia. This case had a posttherapy cytodifferentiation and aggressive clinical course *Note.* Adapted from “Embryonal Rhabdomyosarcoma with Posttherapy Cytodifferentiation and Aggressive Clinical Course,” by Jeyaraju M, Macatangay RA, Munchel AT, York TA, Montgomery EA, Kallen ME. Case Rep Pathol. 2021;2021:1800854 (https://www.hindawi.com/journals/cripa/2021/1800854/). Copyright © 2021 Maniraj Jeyaraju et al.

The role of Bcl-2 as a biomarker in rhabdomyosarcoma has been the subject of several review articles [16, 93, 94] and there are recent papers trying to correlate levels of Bcl-2 and sensitivity to Nab-paclitaxel [86] in rhabdomyosarcoma and ES. New studies should emerge in a short period of time. The Figure 3 represents a rhabdomyosarcoma without changes after conventional treatment.

Previously, in 2015, a study by Olsen et al. [95] demonstrated that the use of navitoclax (a Bcl-2 family antagonist) was able to sensitize neuroblastoma and ES cell lines to chemotherapy agents, via apoptosis due to Bax activity, especially in N-myc amplified neuroblastoma.

These results should be confirmed using more complex models to ensure the anti-tumoral activity and assess toxicity, namely hepatic, but they pave the way for advanced trials with this co-treatment in pediatric cancer.

## Other sarcomas

Despite variable expression of Bcl-2 in soft tissue sarcomas, the anti-Bcl-2 therapy has been studied with promising results. A study from 2020 by Muenchow et al. [96] explored the synergistic association of venetoclax with the proteasome inhibitor bortezomib (BZB). This experiment was based on previous studies that showed that using a proteasome inhibitor stabilized BOK, preventing its ubiquitination and inducing apoptosis [97].

The study by Muenchow et al. [96] was based on tumor cell lines, derived from patients representing six different subtypes of sarcomas: SS, leiomyosarcoma, liposarcoma, rhabdomyosarcoma, chondrosarcoma and osteosarcoma.

The tumor cells had a frequent expression of Bcl-2 and had different p53 status (mutant and wildtype). The study demonstrated a viability reduction of the cells and an induction of apoptotic cell death after therapy with venetoclax and BZB. This effect was mainly due to the accumulation of BOK and the BH3-only protein NOXA, which inhibit the MCL-1 protein (anti-apoptotic). This is a very interesting study since it showed the efficacy of the therapy in different types of sarcomas and was independent of p53 status, with a promising approach for the clinical scenario.

The genetic background is a major player in this setting, since some alterations such as chromosomal rearrangement, *WT1*, and isocitrate dehydrogenase 1 and 2 (*IDH1/2*) point mutations have been associated with high sensitivity to venetoclax [98, 99]. The IDH1/2 mutations are very interesting in sarcomas since they are a hallmark of chondrosarcomas and negatively impact overall survival [100]. Due to the resistance of chondrosarcomas to conventional chemotherapy and radiotherapy [101], the IDH1/2 mutations could represent a viable option for Bcl-2 inhibitors. Co-therapy may also be a possible solution, using the BH3 mimetic ABT-737, capable of Bcl-2 and Bcl-xL inhibition, rendering the chondrosarcoma sensitive to chemotherapy (doxorubicin or cisplatin) regimens, according to the findings of van Oosterwijk et al. [102], in which they described the high expression of Bcl-2 and Bcl-xL in the mesenchymal, clear cell, and dedifferentiated chondrosarcoma subtypes. Recently, the role of microRNA (miRNA) in regulating Bcl-2 families has been unveiled. Veys et al. [103], in 2021, discovered that Bcl-2 was a direct target of miR-342-5p and that Bcl-xL was a direct target of miR-342-5p and miR-491-5p. The study found that, under hypoxia, miR-342-5p decreased the expression of Bak, and miR-491-5p was associated with Bak upregulation. These findings make miRNA a possible and valuable tool for therapy and shifting resistant chondrosarcomas to more sensitive forms.

Interestingly, in osteosarcomas, there were reports that Bcl-2 could be modulated by miRNA expression, namely miR-143. A study from 2010 [104] showed that miR-143 was downregulated in osteosarcoma cell lines and tumor samples, and its levels of restoration allowed a direct targeting of Bcl-2, reducing cell viability and promoting apoptosis. The interaction of miRNA and Bcl-2 was further studied in 2015 [105], where hypoxia was associated with low levels of miR-15a, promoting invasion and migration of tumor cells. The overexpression of miR-15a was found to repress the tumor cells’ invasion and migration via regulation of Bcl-2 and mitochondrial membrane potential. More recently, miR-190b has been shown to have a capacity for inhibiting tumor cell proliferation and promoting apoptosis via Bcl-2 regulation [106]. Pan-inhibitors of Bcl-2 have also been tested in animal models with promising results [107].

Similarly to other sarcoma subtypes, some studies have focused on Bcl-xL and the sensitization of osteosarcoma cell lines to chemotherapy [108], with the use of WEHI-539 (a Bcl-xL selective BH3 mimetic) obtaining a potent increase in apoptosis even with low doses of doxorubicin [109].

Still, in osteosarcomas, a study from 2020 [110] investigated the effects of rapamycin on the apoptosis and proliferation of osteosarcomas cell lines. They found that rapamycin, especially when administered with a Beclin-1 plasmid transfection group, demonstrated an increase of the pro-apoptotic factor Bax and low levels of the anti-apoptotic Bcl-2, thus decreasing the viability of tumor cells and promoting apoptosis. Some studies have focused on the effects of anti-angiogenic drugs, such as apatinib, which exert its effects via vascular endothelial growth factor receptor-2 (*VEGFR2*). A project from Liu et al. [111] on tumor samples (human samples and xenograft models) found that apatinib showed osteosarcoma growth due to inhibition of VEGFR2 and consequently suppressing the STAT3/Bcl-2 signalling pathway, ultimately with low Bcl-2 levels, inducing autophagy and apoptosis. These findings reinforce the role of Bcl-2 in sarcoma progression and therapeutic target.

**Figure 4:**
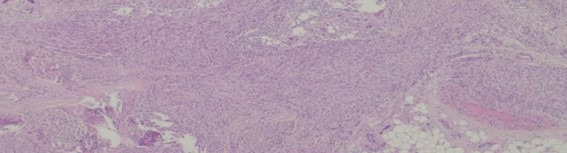
Leiomyosarcoma histological appearance. These tumors are rather resistant to conventional chemotherapy *Note.* Adapted from “Long-Term Response after 94 Cycles of Trabectedin in a Patient with Metastatic Leiomyosarcoma of the Lower Extremity,” by Cordeiro M, Casanova JM, Rodrigues J, Freitas J, Fonseca R, Caetano de Oliveira R, et al. Case Rep Oncol. 2020;13:113–9 (https://karger.com/cro/article/13/1/113/95231/Long-Term-Response-after-94-Cycles-of-Trabectedin). CC-BY-NC.

Leiomyosarcomas are malignant tumors with smooth muscle differentiation [7]. In patients with advanced tumors, there are few therapeutic options, and overall survival is variable according to location—from 15% to 60% [112]. Leiomyosarcomas have a low response to chemo and radiotherapy [113] and despite some good results with trabectedin [114, 115] there are still from the ideal. An example of leiomyosarcoma can be seen in Figure 4.

Marieke de Graaff and colleagues [116] explored the immunohistochemical expression of Bcl-2, Bcl-xL, and Bcl-W and found a high expression of these proteins in leiomyosarcomas, not related to histological grade. Based on the high expression of the proteins in most leiomyosarcomas, they explored the response to doxorubicin and ABT-737, and a synergistic effect was assessed, with cell viability diminution and an increase in apoptosis. The data showed an increased sensitivity of leiomyosarcoma to doxorubicin, due to the ABT-737 administration. Of note, in this study, there was also a relatively high expression of Bcl-2, Bcl-xL, and Bcl-W by immunohistochemistry in myxofibrosarcomas and undifferentiated pleomorphic/spindle cell sarcomas, which may prompt similar studies in these entities.

## Conclusions

The inhibition of members of the Bcl-2 family has been evolving and now encompasses a wider range of tumors and is no longer limited to hematological malignancies. The available data supports the use of these therapies in sarcomas, both in the soft tissues and in the bone, especially when used in combination. The co-treatments regimens are promising since they sensitize the tumors to standard chemotherapy regimens, with good biosafety profile. This possibility will be of extreme value, especially in high-grade sarcomas and in chemo resistant subtypes. Studies with more complex models and clinical trials are essential to assess the clinical translational possibilities.

## Abbreviation

BAK: B-cell lymphoma 2 homologous antagonist/killer
BAX: B-cell lymphoma 2-associated X protein
Bcl-2: B-cell lymphoma 2
BCL-W: B-cell lymphoma 2-like protein 2
BCL-xL: B-cell lymphoma-extra large
BH3: B-cell lymphoma 2 homology 3
BOK: B-cell lymphoma 2 related ovarian killer
ES: Ewing sarcoma
IDH1/2: isocitrate dehydrogenase 1 and 2
MCL-1: myeloid leukemia cell differentiation protein
miRNA: microRNA
NOXA: Phorbol-12-myristate-13-acetate-induced protein 1
SFT: solitary fibrous tumor
SS: synovial sarcoma

## Declarations

### Copyright

© The Author(s) 2023.
